# Effect of diabetes mellitus on the outcomes of total ankle arthroplasty: is controlled diabetes mellitus a risk factor?

**DOI:** 10.1186/s13018-023-04110-y

**Published:** 2023-08-29

**Authors:** Gun-Woo Lee, Dong-Min Jung, Woo-Chul Jung, Keun-Bae Lee

**Affiliations:** 1 Department of Orthopedic Surgery, Chonnam National University Hospital, 42 Jebongro, Donggu, Gwangju, 61469 Republic of Korea; 2 Department of Orthopedic Surgery, Chonnam National University Medical School, Gwangju, Republic of Korea

**Keywords:** Diabetes mellitus, Total ankle arthroplasty, Clinical outcomes, Complications

## Abstract

**Background:**

It is still uncertain whether diabetes mellitus (DM) is a risk factor for poor outcomes and increased complications after total ankle arthroplasty (TAA). The objective of this study was to compare clinical outcomes and complication rates of TAA in patients with and without DM.

**Methods:**

This study enrolled patients with symptomatic end-stage ankle osteoarthritis with a minimum follow-up period of 24 months after TAA. A total of 252 patients (266 ankles) were classified into two groups according to the presence of DM: (1) DM group (59 patients, 67 ankles) and (2) non-DM group (193 patients, 199 ankles). We defined controlled diabetes as (1) HbA1c level < 7.0%, or (2) fasting glucose level < 130 mg/dL with HbA1c level ≥ 7.0% for hospitalization period. Clinical outcomes data (Ankle Osteoarthritis Scale, American Orthopedic Foot and Ankle Society ankle-hindfoot score, Short Form-36 Physical Component Summary score, and visual analog scale for pain) were compared preoperatively and at the final follow-up between the two groups. Complications following TAA were also compared between the two groups.

**Results:**

All clinical variables had improved in both groups by the final follow-up (mean follow-up = 77.8 months). There was no significant difference in any clinical variable between the two groups at the final follow-up (*P* > 0.05). Of the 266 ankles, 73 ankles (19 in the DM group, 54 in the non-DM group) developed periprosthetic osteolysis. Although the DM group showed a higher prevalence of aseptic loosening or subsidence, the difference between the two groups was not statistically significant (*P* = 0.236).

**Conclusions:**

In the intermediate-term follow-up, TAA in patients with controlled DM showed clinical outcomes and complication rates comparable to patients without DM. Our results suggest that TAA can be done safely in diabetic patients if the DM is controlled in the perioperative period.

*Level of evidence*: Therapeutic Level III.

## Introduction

Total ankle arthroplasty (TAA) is becoming more popular as a treatment for end-stage ankle arthritis [1–3]. This popularity stems from TAA's satisfactory long-term outcomes and the restoration of joint function [4–7]. However, several factors, including the patient’s characteristics, pre-and postoperative deformity, and etiology of arthritis, can influence the survivorship of TAA [8–14]. Among the patient's characteristics, diabetes mellitus (DM) is an emerging concern and has been known to influence the clinical outcomes and the development of complications in foot and ankle surgery [15, 16].

Various glucose indicators, such as fasting glucose, postprandial glucose, and glycated hemoglobin (HbA1c), have been used to estimate hyperglycemia [17, 18]. There are still no definitive criteria for hyperglycemia to indicate whether orthopedic surgery can be safely performed; the level of HbA1c is commonly used as a possible substitute for fasting glucose because it is a slowly changing value that represents glycemic control over a 3-month period [19]. However, the most appropriate threshold value for defining high HbA1c is still controversial, and it is difficult to achieve a threshold of 7.0% HgA1c in elderly diabetic patients awaiting arthroplasty [20, 21].

Among the few studies that have looked at the impact of DM on the outcomes of TAA [22–24], the results are inconsistent and difficult to compare due to the use of various types of implants or the involvement of multiple surgeons. In addition, a recent study reported that there was little evidence of an association between preoperative glycemic control and postoperative outcomes following hip and knee arthroplasty [25]. So, it is still unclear whether DM is a risk factor for poor outcomes and increased complications after TAA. We hypothesized that the presence of controlled DM during the perioperative period would neither negatively affect the clinical outcomes nor increase postoperative complications of TAA. Therefore, the objective of this study was to compare clinical outcomes and complication rates of TAA in patients with and without DM.

## Methods

### Patients

This study was approved by the Institutional Review Board of our hospital, and patient consent was waived due to the retrospective nature of this study. We enrolled patients who underwent TAA for end-stage ankle arthritis between January 2005 and December 2018. All operations were performed by a single surgeon using mobile-bearing HINTEGRA prostheses (Newdeal, Lyon, France/Integra Lifesciences, Plainsboro, NJ USA).

Our study included patients with symptomatic end-stage ankle osteoarthritis with a minimum follow-up period of 24 months after TAA. Patients with diabetes were identified as those requiring treatment with oral hypoglycemic medications and/or insulin at the time of surgery. Concerning diabetic patients, this study included only patients with controlled diabetes without other diabetic complications. We defined controlled diabetes as (1) HbA1c level < 7.0%, or (2) fasting glucose level < 130 mg/dL with HbA1c level ≥ 7.0% for hospitalization period before and after TAA.

Exclusion criteria were secondary osteoarthritis due to osteonecrosis of the talus, rheumatoid arthritis, hemophilic arthropathy, previous history of infection, and takedown of ankle arthrodesis. Finally, a total of 252 patients (266 ankles) were enrolled and classified into two groups according to the presence of DM: (1) DM group (59 patients, 67 ankles) and (2) non-DM group (193 patients, 199 ankles). The demographic data for these patients are shown in Table 1.

**Table 1:** Patient demographic characteristics

	DM Group (N = 67 ankles)	non-DM Group (N = 199 ankles)	P value*
Age (year)†	65.5 ± 7.7 (41 to 83)	63.8 ± 9.0 (31 to 86)	0.166
Sex (year) ‡			0.356
Male	32 (47.8%)	108 (54.3%)	
Female	35 (52.2%)	91 (45.7%)	
BMI (kg/m^2^)†	26.2 ± 2.9 (19.8 to 35.2)	25.8 ± 3.3 (18.8 to 39.5)	0.407
Current smoker‡	9 (13.4%)	33 (16.6%)	0.541
HbA1c (%)	6.8 ± 1.0 (5.1 to 8.9)	–	–
Diagnosis‡			0.077
Primary osteoarthritis	37 (55.2%)	80 (40.2%)	
Posttraumatic			
Fracture	10 (14.9%)	49 (24.6%)	
Recurrent ankle sprain	20 (29.9%)	70 (35.2%)	
Hospital stay (days)†	16.6 ± 4.4 (7 to 27)	15.8 ± 4.0 (7 to 28)	0.231
Follow-up (months)†	79.8 ± 48.6 (24 to 205)	77.2 ± 42.6 (24 to 207)	0.691

*DM* Diabetes Mellitus, *BMI* body mass index, *HbA1c* glycated hemoglobin

^*^The independent t test was used to analyze age differences in age, BMI, hospital stay, and follow-up duration. The chi-square or Fisher’s exact test was used to analyze differences in sex, smoking status, and diagnosis. A p value of < 0.05 was considered significant

^†^The values are given as the mean ± standard deviation, with the range in parentheses

^‡^The values are given as the number of ankles, with the percentage in parentheses

### Surgical technique and postoperative management

In all patients, primary TAA was performed under general anesthesia using a longitudinal anterior approach between the tibialis anterior and extensor hallucis longus tendons. Standard TAA procedures such as bone resection and implantation were subsequently carried out. If necessary, concomitant procedures for osseous alignment or soft-tissue balancing were performed during the index surgery or subsequently. At 4 weeks after surgery, patients were instructed to begin increasing weight-bearing while wearing an ankle–foot orthosis. At 6–8 weeks following surgery, full weight-bearing ambulation without an orthosis or ambulation aids was allowed. Patients were followed-up at 1, 3, 6, and 12 months postoperatively and annually thereafter.

### Clinical evaluation

Clinical evaluation was based on preoperative and final follow-up assessments. The pain and disability subscores of the Ankle Osteoarthritis Scale (AOS) [26], American Orthopedic Foot and Ankle Society (AOFAS) ankle-hindfoot score [27], the Short Form-36 Physical Component Summary (SF-36 PCS) score [28], and the visual analog scale (VAS) score for pain were included as clinical assessment tools. The AOS was chosen as the primary assessment tool, because of its reliability and validity for ankle arthritis [29]. It was also important to determine whether the AOS had improved beyond the minimal clinically relevant difference (MCID), which refers to the change in patients who described themselves as minimally better or minimally worse as a result of the surgery [30]. All clinical outcomes were evaluated by two blinded, independent observers who were not directly involved in the surgical procedures.

Complications following surgery were classified as either "major" or "minor" complications. Deep infection and periprosthetic osteolysis were major complications, while wound problems and heterotopic ossification were minor complications. The wound problems were classified into two categories: wound dehiscence and allergic reaction. The wound dehiscence is defined as a partial or complete separation of an incision wound due to a failure of proper wound healing. The allergic reaction was defined as an occurrence of eczematous eruption or blister around the incision wound [31].

### Statistical analyses

Standard methods were employed to calculate descriptive statistics, and the Kolmogorov–Smirnov test was utilized to check for normality. The independent t test was used to analyze differences between groups in normally distributed continuous data. Differences in dichotomous variables were analyzed using the chi-square test or Fisher exact test. Data were analyzed using SPSS (version 23.0, IBM Corporation, Armonk, NY USA). All statistical analyses were reviewed by a statistician, and a *P* value < 0.05 was considered to be statistically significant.

## Results

### Clinical outcomes

Clinical outcomes are summarized in Table 2. The mean preoperative AOS pain scores were 54.9 in the DM group and 56.4 in the non-DM group, and these scores improved to 18.7 and 21.8, respectively, at final follow-up. The mean preoperative AOS disability score was 69.1 in the DM group and 66.7 in the non-DM group, and these scores improved to 29.7 and 29.5, respectively, at final follow-up. Both groups had clinical outcomes that were better than the MCID criterion for AOS (28.0 points). The AOFAS ankle-hindfoot score, SF-36 PCS, and VAS for pain improved in both groups at final follow-up. However, there was no significant difference in any clinical variable between the two groups at final follow-up (*P* > 0.05).

**Table 2:** Comparison of clinical outcomes between the DM Group and non-DM group following total ankle arthroplasty

	DM Group (N = 67 ankles)	Non-DM Group (N = 199 ankles)	P value*
AOS pain^†^			
Preoperative	54.9 ± 15.4 (21.4–82.9)	56.4 ± 15.1 (2.9–92.9)	0.533
Final	18.7 ± 16.8 (0.0–68.9)	21.8 ± 15.8 (0.0–70.0)	0.214
AOS disability^†^			
Preoperative	69.1 ± 12.4 (43.3–93.3)	66.7 ± 14.2 (38.9–98.9)	0.294
Final	29.7 ± 19.4 (2.2–78.6)	29.5 ± 16.7 (0.0–80.0)	0.917
AOFAS ankle-hindfoot score^†^			
Preoperative	51.4 ± 12.5 (21.0–84.0)	50.2 ± 13.0 (14.0–87.0)	0.551
Final	85.2 ± 12.7 (44.0–100)	82.5 ± 11.4 (46.0–100)	0.147
SF-36 PCS score^†^			
Preoperative	46.2 ± 15.9 (19.5–77.3)	43.0 ± 15.4 (10.0–85.5)	0.285
Final	65.9 ± 20.2 (20.0–94.3)	68.4 ± 18.3 (19.5–97.0)	0.456
VAS pain^†^			
Preoperative	6.5 ± 1.5 (4.0–9.0)	6.8 ± 1.5 (3.0–9.0)	0.279
Final	2.1 ± 1.9 (0.0–6.0)	2.7 ± 1.9 (0.0–7.0)	0.214

*DM* Diabetes Mellitus, *AOS* Ankle Osteoarthritis Scale, *AOFAS* American Orthopedic Foot and Ankle Society, *SF-36 PCS* Short Form-36 Physical Component Summary, *VAS* Visual Analogue Scale

^*^The independent t test was used to analyze differences in AOS pain, AOS disability, AOFAS ankle-hindfoot score, SF-36 PCS scores, and VAS pain scores. *p* < 0.05 was considered significant

^†^Numbers represent the mean and standard deviation, with the range in parentheses

### Complications

The numbers of overall, major, and minor complications were not significantly different between the two groups (Table 3). Of the 266 ankles, 73 ankles (19 in the DM group, 54 in the non-DM group) developed periprosthetic osteolysis. Among them, 9 ankles (4 in the DM group, 5 in the non-DM group) with aseptic loosening or subsidence of the component were revised to arthrodesis or revision TAA with exchanging component. For the other 64 ankles, 29 ankles (9 ankles in the DM group, 20 in the non-DM group) were treated by bone grafting for osteolysis that has progressive and/or symptomatic features. Although the DM group showed a higher prevalence of aseptic loosening or subsidence, we did not find a statistically significant difference between the two groups (*P* = 0.236).

**Table 3:** Complications associated with total ankle arthroplasty according to the presence of DM

	DM Group (N = 67 ankles)	Non-DM Group (N = 199 ankles)	*P* value*
Major†	20 (29.9%)	55 (27.6%)	0.728
Deep infection	1 (1.5%)	1 (0.5%)	0.442
Periprosthetic osteolysis			
Alone	15 (22.4%)	49 (24.6%)	0.711
With aseptic loosening/subsidence	4 (6.0%)	5 (2.5%)	0.236
Minor†	24 (35.8%)	74 (37.2%)	0.841
Wound Problem	2 (3.0%)	1 (0.5%)	0.157
Heterotopic Ossification	22 (32.8%)	73 (36.7%)	0.570
Total (no. [%])	44 (65.7%)	129 (64.8%)	0.900

*DM* Diabetes Mellitus

^*^The chi-square or Fisher’s exact test was used to analyze differences in the number of complications. A p value of < 0.05 was considered significant

^†^The values are given as the number of ankles, with the percentage in parentheses

Regarding the surgical site infection, each of the two groups had one case of deep infection after TAA. These two ankles were successfully treated by revision TAA in a two-stage procedure using an antibiotics-impregnated cement spacer. Three cases of wound problems included 2 cases of wound dehiscence with partial breakdown (1 in the DM group and 1 in the non-DM group) and 1 case of an allergic reaction (1 in the DM group). Patients with wound dehiscence were healed with additional wound closure and prolonged antibiotic use. One case of allergic reaction was successfully treated with topical antibiotic ointment and foam dressing. The non-DM group showed a higher prevalence of heterotopic ossification after TAA than the DM group; however, the difference between the two groups was not statistically significant (*P* = 0.570).

## Discussion

Orthopedic surgeons often have difficulty deciding whether to operate on diabetic patients to minimize complications and obtain satisfactory results. This is one of the largest studies to date that have evaluated the effects of DM on the outcomes of TAA. The strength of this study was that it evaluated consecutive patients with osteoarthritis having a single type of implant. The results of this study indicate that TAA can be safely performed in diabetic patients when DM is well controlled during the perioperative period, even if HbA1c is slightly high than normal. We also found that DM did not negatively affect clinical outcomes or increase complication rates.

Several previous studies have investigated the effect of DM on the outcomes of foot and ankle surgeries. SooHoo et al. found that DM was a strong risk factor associated with open reduction and internal fixation of ankle fractures [16]. They reported that diabetic patients likely have severe complications including infection and amputation. Schmidt et al. found that patients with diabetes experienced higher rates of postoperative complications, particularly deep infection after fixation of ankle fractures [32]. They also showed that diabetic patients were more likely to require unplanned secondary surgical procedures including debridement, arthrodesis, and amputation. However, they found that clinical outcomes were not significantly affected.

Previous studies have evaluated the impact of DM on outcomes of TAA. Choi et al. investigated 173 ankles, including 43 ankles of diabetic patients, and reported that DM negatively affected clinical outcomes [23]. They also found that the incidence of early onset osteolysis was significantly higher in the diabetic group than in the non-diabetic group (*P* = 0.02). However, Gross et al. reported that diabetic patients had similar complication rates, revision rates, and functional outcomes compared with a control group [22]. In the present study, we achieved satisfactory clinical outcomes and complication rates for the DM group, which were comparable to those for the non-DM group. In this study, although the DM group showed a higher prevalence of aseptic loosening or subsidence of implant than the non-DM group, this difference was not statistically significant (*P* = 0.236). There was no significant intergroup difference in the prevalence of overall osteolysis cases (19 in the DM group, 54 in the non-DM group, *P* = 0.846).

Regarding the wound problem, only 2 patients (3.0%) showed wound problems in the DM group in the present study and there was no significant difference between the two groups (*P* = 0.157). A previous meta-analysis reported that superficial wound healing complications were developed at a mean of 8% (range, 0 – 14.7) by analyzing 827 TAA [7]. However, a recent study analyzed 762 primary TAA and reported that 26 patients (3.4%) had wound issues. Furthermore, wound issues occurred in about 3.1% of patients with DM, and they did not find any meaningful increase in wound complications in those with DM compared to those without DM [33]. Choi et al. reported that the incidence of wound complications was as high as 13.9% (6 of 43) in the DM group, but it was only 4.0% (1 of 25) in the controlled DM group [23].

The level of HbA1c, a slowly changing variable that reflects glycemic control over the previous 3 months, is a criterion commonly used by orthopedic surgeons in deciding whether to perform TAA surgery on diabetic patients. While there is still no definitive threshold for patient selection, previous studies have shown that an HbA1c level > 7.0% is associated with increased risk of complications [21, 34–36]. Despite proper diabetic management, however, it is often difficult to achieve an HbA1c level ≤ 7.0% in patients awaiting orthopedic surgery. Giori et al. investigated 404 diabetic patients awaiting total hip or knee arthroplasty and found that an HbA1c level ≤ 7.0% was achieved by only 35 (59.3%) of 59 patients presenting with an HbA1c level > 7.0%, and some patients had to delay surgery to meet the threshold value [20]. Thus, they suggested raising the HbA1c threshold to 8.0% if patients are well on acute glycemic control. In this study, 17 (25.4%) out of 67 patients showed HbA1c levels > 7.0%, and among them, complications occurred only in 6 patients (7 cases: 3 of osteolysis alone, 3 of heterotopic ossification). No cases of deep infection or wound problems were reported.

Our study has several acknowledged limitations. First, this study was retrospective in design and had a relatively small sample size of the DM group. Although we analyzed prospectively collected data and the two groups showed comparable demographic characteristics, these limitations may mitigate the ability to evaluate outcomes on a general topic such as this study. Second, this study included only patients with controlled DM. The number of patients with uncontrolled DM was very small since the majority of patients with uncontrolled DM had delayed TAA. Thus, this study did not perform a further evaluation of the uncontrolled DM group. Further studies are needed to evaluate the effect of uncontrolled DM on the outcomes of TAA. Finally, this study defined controlled DM based on the level of HbA1c and fasting glucose. Despite being based on previous studies, there is still no clear definition of controlled DM in elective orthopedic surgery. In addition, DM control was judged based on a hospitalization period.

In conclusion, TAA in patients with controlled DM showed comparable clinical outcomes and complication rates relative to those without DM on intermediate-term follow-up. Our results suggest that TAA can be safely performed in patients with DM if the DM is controlled in the perioperative period. However, these findings should be taken with caution and further prospective studies with a large sample size are necessary to validate our results.

## Data Availability

The datasets analyzed during the current study are not publicly available due to patient confidentiality.
